# Detection of Low-Frequency *KRAS* Mutations in cfDNA From *EGFR*-Mutated NSCLC Patients After First-Line EGFR Tyrosine Kinase Inhibitors

**DOI:** 10.3389/fonc.2020.607840

**Published:** 2021-01-15

**Authors:** Giorgia Nardo, Jessica Carlet, Ludovica Marra, Laura Bonanno, Alice Boscolo, Alessandro Dal Maso, Andrea Boscolo Bragadin, Stefano Indraccolo, Elisabetta Zulato

**Affiliations:** ^1^ Immunology and Molecular Oncology Unit, Istituto Oncologico Veneto IOV - IRCCS, Padova, Italy; ^2^ Medical Oncology 2, Istituto Oncologico Veneto IOV - IRCCS, Padova, Italy; ^3^ Department of Surgery, Oncology and Gastroenterology, University of Padua, Padova, Italy

**Keywords:** *KRAS*, *EGFR*, liquid biopsy, non-small-cell lung cancer, cell free DNA, tyrosine kinase inhibitors

## Abstract

**Background:**

Molecular profiling of advanced *EGFR* mutated NSCLC has recently demonstrated the co-existence of multiple genetic alterations. Specifically, co-existing *KRAS*-mutations in *EGFR* NSCLCs have been described, despite their prevalence at progression and their role in the response to *EGFR* tyrosine kinase inhibitors (TKIs) remain marginally explored. Aim of our study was to investigate the prevalence of co-existing *KRAS* mutations at the time of progressive disease and explore their impact on clinical outcome.

**Materials and Methods:**

We retrospectively analyzed by digital droplet PCR prevalence of *KRAS* co-mutations in 106 plasma samples of *EGFR* mutated NSCLC patients, in progressive disease after *EGFR* TKI treatment as first-line therapy.

**Results:**

*KRAS* co-mutations (codon 12 and 13) were identified in 3 patients (2.8% of analyzed samples), with low allelic frequency (<0.2%), and had a negative impact on clinical outcome to first-line *EGFR* TKI.

**Conclusion:**

Detection of *KRAS* mutations in cell-free DNA of *EGFR* mutant NSCLC patients at progression after first or second generation *EGFR* TKI is a rare event. Due to their low abundance, the negative impact of *KRAS* mutations on the response to *EGFR* TKI remains to be confirmed in larger studies.

## Introduction

Non-small-cell lung cancer (NSCLC) is the leading cause of cancer-related mortality worldwide with five-year survival rate less than 10% among patients with advanced disease (1). Activating mutations in the epidermal growth factor receptor (*EGFR*) gene occur as early cancer-driving clonal event (2) in a subset of NSCLC patients (approximately 15% of Caucasian patients) and predict sensitivity to *EGFR* tyrosine kinase inhibitors (TKIs) (3). Improvement in clinical outcome, in terms of objective response rate (ORR), progression-free survival (PFS) and overall survival (OS), compared with upfront platinum doublet chemotherapy, made TKIs standard of care for advanced stage *EGFR* mutant NSCLC (4–8). However, resistance invariably develops, with EGFR T790M mutation accounting for approximately 50–60% of the mechanisms of acquired resistance to first- or second-generation *EGFR*-TKI therapy (3). Other less common *EGFR*- independent mechanisms of resistance include activation of bypassing pathways and histologic transformation to small-cell lung cancer (10–15% of cases) (9–11). In addition, 20–30% of patients do not show response on *EGFR* TKI treatment, probably due to intrinsic mechanisms of resistance (12).

Recently, comprehensive molecular-pathological profiling of advanced *EGFR* mutated NSCLC prior to therapy demonstrated co-existence of multiple genetic alterations (13). Consequently, the question arises as to whether co-occurring genetic alterations cooperate with the primary driver *EGFR* gene in promoting tumor progression and limiting efficacy of target therapy. Recent studies showed that co-mutations in the *TP53* gene are a negative predictive factor of response to *EGFR*-TKI and an independent prognostic factor of shorter survival in advanced *EGFR* mutant NSCLC (14–17). Moreover, co-existing *KRAS*-mutations in *EGFR* NSCLCs have been reported by several studies (18–23). However, their prevalence at progression and their role in the response to TKIs treatment has been investigated only in one study including a small number of patients (n=33) (24).

Here, we performed a retrospective analysis of *KRAS* co-genetic alterations in 106 *EGFR* mutated NSCLC patients with progressive disease after *EGFR* TKI first-line therapy. We quantitated *KRAS* mutation in plasma samples by droplet digital PCR (ddPCR), with the aim to investigate the prevalence of co-existing *KRAS* mutations at the time of progression and explore their impact on clinical outcome.

## Material and Methods

### Study Design and Patient Population

The primary aim of this study was to assess the prevalence of *KRAS* co-mutations in *EGFR* mutated NSCLC patients, in progressive disease after *EGFR* TKI treatment as first-line therapy. For this purpose, we retrospectively selected 122 consecutive patients with *EGFR*-mutated NSCLC with progressive disease after first-line TKI treatment, referring to our Institution from 2016 to 2019. Eligibility criteria were confirmed histological diagnosis of advanced NSCLC, presence of an *EGFR* exon 18 to 21 mutation at diagnosis, progression to front line systemic treatment with first- or second- generation *EGFR* TKIs (erlotinib, gefitinib, or afatinib), and available liquid biopsy material collected at progressive disease, and clinical follow-up. Patients who did not progress to first-line *EGFR* TKIs, or without available liquid biopsy material after progression were excluded.

At the time of diagnosis, tissue molecular analyses of *EGFR* gene exons 18 to 21 were performed according to standard clinical practice, and *KRAS* mutational status was not routinely examined because mutually exclusive with activating *EGFR* mutations in this patient population.

At progressive disease, plasma samples were collected for liquid biopsy to assess the T790M mutational status in cell-free (cf)-DNA. Molecular analyses were performed according to standard lab practice, using the CE IVD cobas® EGFR Mutation Test v2.

The studies involving human participants were reviewed and approved by IOV Institutional Review Board and Ethics Committee (CESC IOV 2020/57), and were performed in accordance with the declaration of Helsinki. The patients/participants provided their written informed consent to participate in this study. For patients who were dead or lost to follow-up at the time of study enrolment, we used the Italian Data Protection Authority Authorisation 9/2016 on “privacy protective rules for recording clinical data for research and study purposes”.

### Cell Free DNA (cfDNA) Extraction and Analysis

Residual plasma collected at the time of progression for routine diagnostic activity was used: cfDNA was extracted from 1–2 ml of plasma using the Maxwell® RSC ccfDNA Plasma Kit (Promega, Madison, Wisconsin, USA), and eluted into 60 µl of buffer, according to manufacturer’s instructions. cfDNA was quantified using the QuBit dsDNA HS Assay kit with QuBit 3.0 fluorimeter (Thermo Fisher Scientific, San Jose, CA), and stored at −20°C before use.

Detection of *KRAS* mutations in codons 12 and 13 in cfDNA was performed by droplet digital PCR (ddPCR), as previously described (25). The ddPCR assay was purchased from Bio-Rad (the ddPCR *KRAS* G12/G13 Screening Kit #186-3506), and it does not enable to distinguish among different mutations in *KRAS* codon G12/G13 (G12A, G12C, G12D, G12R, G12S, G12V, G13D). Each sample was analyzed in triplicate and in each test at least three control wells with a negative *KRAS* cfDNA, one negative control well without DNA and one positive control were included. In line with our previous study (25) and as reported in the manufacturer’s instructions, a cut-off of three droplets was used to call a sample mutant, according to the Poisson’s law of small numbers. The sensitivity of our assay to detect *KRAS* mutation in plasma samples was 48% (25).

### Data Analysis

Progression free survival (PFS) was calculated as the time between the first day of treatment and the radiologic and/or clinical evidence of progression; time to treatment failure (TTF) was defined as the time from the first day of *EGFR*-TKI administration to the date of treatment failure; overall survival (OS) was measured as the time elapsed from diagnosis to death for any cause. Patients who did not develop an event during the study period were censored at the date of last observation. Median PFS, TTF and OS were estimated using the Kaplan–Meier method.

Chi-square test was used to evaluate whether the frequency of cases with single or double *KRAS* positive droplets differ among *EGFR* mutant and *EGFR* wild-type cfDNA samples.

## Results

### Patients

**Figure 1:**
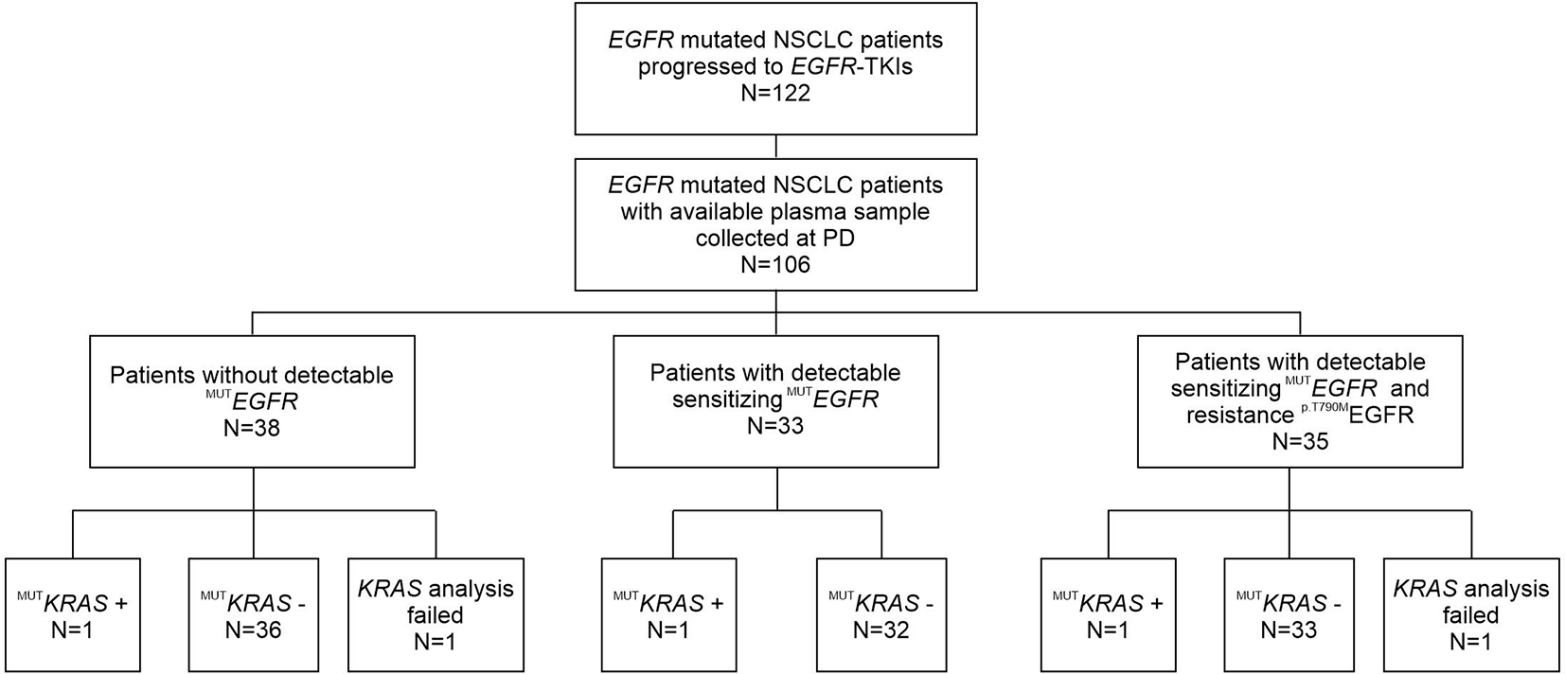
Flow chart of patients enrolled in this study, who progressed after front-line first- or second-generation *EGFR*-TKI treatment and underwent liquid biopsy to assess the T790M mutational status.

Study layout is summarized in **Figure 1**. From 2016 to 2019, 122 patients with advanced *EGFR* mutated NSCLC referring to our Institution received treatment with a first- or second -*EGFR*-TKI as first-line therapy and underwent cfDNA genotyping for assessment of *EGFR* mutations at progression. Residual plasma samples were available for 106 patients. Clinical characteristics of patients matching the eligibility criteria are shown in **Table 1**. At the time of diagnosis, median age was 68 years. Most patients were females (59%), with *EGFR* mutant stage III–IV lung adenocarcinoma (90.5%) and without smoking history (62%). Patients presented in an optimal or good Eastern Cooperative Oncology Group (ECOG) performance status (PS), with 46 (43%) and 55 (52%) having ECOG 0 and 1, respectively. *EGFR* exon 19 deletion was carried by 64 out of 106 patients (60%); 35 patients (33%) had an EGFR p.L858R point mutation and 7 (7%) had different *EGFR* mutations. The majority of patients (n=54) received gefitinib as first-line TKI treatment (51%), 26 out of 106 (24.5%) patients received erlotinib, and 26 (24.5%) afatinib (**Table 1**). Median Progression Free Survival (PFS) was 24.30 months (95%, CI: 19.29–29.31).

**Table 1:** Clinical features of patients at time of diagnosis.

**Age at diagnosis (years)**	Median (Q1–Q3)	68 (35–85)
**Gender**	Male	43 (41%)
** **	Female	63 (59%)
**Smoking**	No	66 (62%)
	Yes	7 (7%)
	Ex	28 (26%)
** **	nd	5 (5%)
**PS**	0	46 (43%)
	1	55 (52%)
	2	3 (3%)
** **	nd	2 (2)
**Stage at diagnosis**	I–II	10 (9.5%)
** **	III–IV	90 (90.5%)
**Baseline EGFR mutation status**	Exon 19 deletion	64 (60%)
	Ex 21 mutations (L858R)	35 (33%)
** **	Others	7 (7%)
**Type of treatment**	Gefitinib	54 (51%)
	Erlotinib	26 (24.5%)
	Afatinib	26 (24.5%)
**Best response to TKI**	CR	1 (1%)
	PR	71 (67%)
	SD	18 (17%)
	PD	11 (10%)
	nd	5 (5%)
**T790M status at progressive disease**	T790M positive	35 (33%)
	T790M negative	71 (67%)
**PFS (months)**	Mean (CI 95%)	24.30 (19.29–29.31)
**TTF (months)**	Mean (CI 95%)	41.44 (29.45–53.39)
**OS (months)**	Mean (CI 95%)	67.98 (51.07–84.89)
**Total**		**106**

nd, no determined.

At the time of progressive disease, *EGFR* sensitizing mutations were detected by liquid biopsy in 68 out of 106 plasma samples tested (64%), whereas the remaining 38 plasma samples (36%) did not bear *EGFR* mutations (**Table 2**). The T790M-resistance mutation was found in 35 out of 106 samples (33%), or 35 out of 68 plasma samples bearing *EGFR* sensitizing mutations (50.7%) (**Table 2**).

**Table 2:** *EGFR* mutational status.

Diagnosis*	n° (%)	Progressive Disease**	n° (%)
			
** Exon 19 deletion**	64 (60 %)	No detectable mutations	19 (29.7 %)
		Exon 19 deletion +	19 (29.7 %)
		Exon 19 deletion + and T790M +	26 (40.6 %)
** Exon 21 mutation (L858R)**	35 (33 %)	No detectable mutations	13 (37.1 %)
		Exon 21 mutation +	13 (37.1 %)
		Exon 21 mutation + and T790M +	9 (25.8 %)
** Other mutations**	7 (7 %)	No detectable mutations	6 (85.7 %)
		EGFR-sensitizing mutation +	1 (14.3 %)
		Mutation + and T790M +	0 (0 %)

*Tissue (FFPE); **cfDNA.

### Prevalence of *KRAS* Co-Mutations at Progressive Disease

Among 106 patients with plasma samples available, 104 were successfully screened by ddPCR for the presence of concomitant *KRAS* mutation in codon 12 and 13, whereas 2 samples were not evaluable (**Figure 1**).

**Figure 2:**
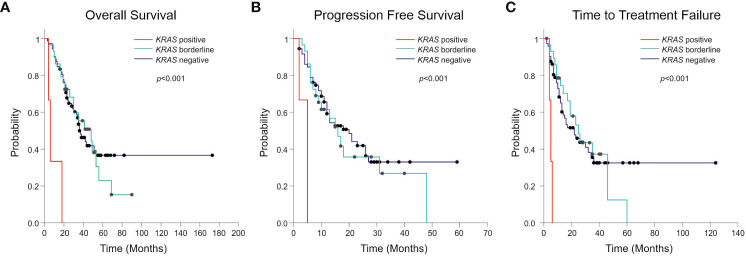
Kaplan-Meier curves showing Overall Survival (OS) **(A)**, Progression Free Survival (PFS) **(B)** and Time to Treatment Failure (TTF) **(C)** according to the presence, the absence, or borderline positivity (1–2 positive droplets) of *KRAS* mutation. The *p*-value related to the presence or the absence of *KRAS* mutation is reported in figure.

Considering the standard cut-off value of three droplets, as detailed in the *Materials and Methods* section, *KRAS* mutations were detected in 3 patients (2.8%) (**Figure 1**). Tumor tissue collected at diagnosis was available only for one (ID#88) out of 3 *KRAS* positive patients, and its analysis confirmed the co-existence of *EGFR* and *KRAS* mutations (*KRAS* allelic frequency 13.8%). In all 3 positive cases, the allelic frequency of the *KRAS* mutations in the liquid biopsy samples was low (<0.2%) (**Table 3**). All *KRAS* positive patients (n=3) had poor clinical outcome to first-line *EGFR* TKI, in terms of TTF, PFS and OS (**Figure 2** **;** **Table 3**). Interestingly, these patients were current or former smoker and one of them had squamous cell carcinoma histology. At diagnosis they all presented with extra-thoracic disease, but they did not show any specific clinical negative prognostic marker (i.e. worse performance status; see **Table 3**). Two of them did not respond to first line *EGFR* TKI, while one of them achieved partial response with a PFS of about six months. At progression to first line TKI, only one of them carried T790M mutation (ID#39), but he did not respond to osimertinib.

**Table 3:** *EGFR* and *KRAS* co-mutated cases.

Patients ID	Diagnosis -Sensitizing ^MUT^ *EGFR*	Progression Disease - ^MUT^ *EGFR* in liquid biopsy	*KRAS* n° of positive droplets (MAFA)	Age at diagnosis (years)	Smoking	PS	Stage at Diagnosis	PFS (months)	TTF (months)	OS (months)
88	L858R	no mutation	4 (0.11%)	77	yes	1	IV	2	4	4
13	ex19 del	ex19 del	3 (0.11%)	65	ex	1	IV	5	6	18
39	ex19 del	ex19del-T790M	3 (0.036%)	71	yes	0	IV	5	5	6

Interestingly, one or two positive droplets for *KRAS* mutations were detected in additional 28 plasma samples out of 104 analyzed (27%), with allelic frequency of the *KRAS* variant very low (mean 0.15%; median 0.12%) and ranging between 0.016 and 0.32% (**Table 4**). These single or double *KRAS* positive droplets were similarly distributed among *EGFR* mutant and *EGFR* wild-type cfDNA samples (15 out of 68 *EGFR* mutant versus 13 out of 38 *EGFR* wild-type samples, respectively. Chi-square test, *P*=0.21). With regard to clinical correlates, patients with borderline *KRAS* positivity (n=28) behave similarly to *KRAS* negative patients (n=73) in terms of TTF, PFS, and OS (**Figure 2**). Details about median TTF, PFS and OS in *KRAS* positive, borderline and negative patients are reported in **Table 5**.

**Table 4:** *KRAS* borderline samples with one or two positive droplets in cfDNA.

Patient ID	Diagnosis- Sensitizing ^MUT^ *EGFR*	Progression Disease - ^MUT^ *EGFR* in liquid biopsy	*KRAS* n° of positive droplets (MAFA)	Age at diagnosis (years)	Smoking	PS	Stage at Diagnosis	PFS (months)	TTF (months)	OS (months)
16	L858R	no mutation	1 (0.12%)	72	no	1	IV	11	21	21
20	ex19 del	no mutation	2 (0.32%)	73	no	0	IV	28	33	39
54	ex19 del	no mutation	1 (0.10%)	85	\	1	IVb	48	60	69
56	L858R	no mutation	1 (0.10%)	49	no	0	IVb	3	9	9
57	G719	no mutation	1 (0.30%)	63	ex	0	Ib	10	19	53
78	ex19 del	no mutation	1 (0.19%)	71	\	0	IVa	31	46	48
81	ex19 del	no mutation	1 (0.22%)	72	no	0	IVa	13	35	49
92	ex19 del	no mutation	1 (0.30%)	75	ex	0	IV	40	41	51
3	ex19 del	ex19 del	1 (0.24%)	80	no	1	IV	32	40	46
9	ex19 del	ex19 del	2 (0.06%)	67	ex	2	IV	5	8	10
23	ex19 del	ex19 del	1 (0.25%)	60	no	0	IV	8	11	23
29	ex19 del	ex19 del	1 (0.07%)	63	ex	0	IV	9	14	19
33	ex19 del	ex19 del	1 (0.31%)	56	no	1	IV	4	4	4
45	ex19 del	ex19 del	1 (0.07%)	64	no	1	IV	15	17	21
52	ex19 del	ex19 del	1 (0.11%)	67	no	1	IVb	5	5	11
91	ex19 del	ex19 del	1 (0.15%)	55	\	0	Ia	16	46	56
102	ex19 del	ex19 del	1 (0.18%)	68	no	1	IV	9	10	22
48	L858R	L858R	1 (0.29%)	79	no	1	IV	17	25	26
76	L858R	L858R	2 (0.07%)	54	no	1	IIIb	18	19	30
44	L858R	L858R - S768I	1 (0.016%)	44	no	0	Ib	7	9	17
19	ex19 del	ex19 del - T790M	1 (0.03%)	68	yes	0	Iib	10	10	42
27	ex19 del	ex19 del - T790M	1 (0.12%)	37	no	0	IV	10	35	90
30	ex19 del	ex19 del - T790M	1 (0.22%)	51	no		IV	6	23	35
41	ex19 del	ex19 del - T790M	2 (0.24%)	64	ex	0	IV	15	2	44
43	ex19 del	ex19 del - T790M	1 (0.07%)	66	no	1	IV	8	12	30
49	ex19 del	ex19 del - T790M	1 (0.21%)	82	ex	0	IV	22	28	69
60	ex19 del	ex19 del - T790M	1 (0.20%)	60	no	1	IIIa	6	26	41
36	L858R	L858R - T790M	1 (0.16%)	71	no	1	IV	6	7	16

**Table 5:** Overall Survival (OS), Progression Free Survival (PFS) and Time to Treatment Failure (TTF) in *KRAS* negative, borderline and positive patients at Progressive Disease (PD).

***KRAS***	**N°**	**mOS (m)**	**CI95% (m)**	**P-value**
Negative	75	36	28.5-43.5	
Borderline	28	48	23.2-72.8	<0.001
Positive	3	6	2.8-9-2	
				
***KRAS***	**N°**	**mPFS (m)**	**CI95% (m)**	**P-value**
Negative	75	20	12-6-27-4	
Borderline	28	16	10-2-21-8	<0.001
Positive	3	5	5-0-5-0	
				
***KRAS***	**N°**	**mTTF (m)**	**CI95% (m)**	**P-value**
Negative	75	22	11-5-32-5	
Borderline	28	25	14-9-35-1	<0.001
Positive	3	5	3.4-6.6	

mOS, median Overall Survival; mPFS, median Progression Free Survival.

mTTF, median Time To Failure treatment; m, months.

We conclude that frank positivity for codon 12 and 13 *KRAS* mutations in cfDNA of *EGFR* mutant NSCLC at progression after first or second generation *EGFR* TKI treatment is a rare event.

## Discussion

We report a retrospective evaluation of the prevalence of codon 12 and 13 *KRAS* co-mutations in *EGFR* mutated NSCLC patients in progressive disease after *EGFR* TKI treatment as first-line therapy, with the aim to establish their prevalence and explore their impact on clinical outcome. Mutations in *EGFR* and *KRAS* are considered mutually exclusive in NSCLC (26) and this is also remarked by epidemiologic data, being *KRAS* mutations associated with smoke and *EGFR* mutations more common in non-smokers, respectively. On the other hand, genetic studies involving multi-region sequencing of tumors have clearly shown that genetic heterogeneity exists in lung adenocarcinoma and *EGFR* mutations generally occur in the genetic trunk of the tumor and are hence clonal, whereas *KRAS* mutations are often sub-clonal (2). This genetic model is also supported by studies which investigated *EGFR* and *KRAS* mutations in matched primary tumor and metastasis from the same patients and reported the occasional presence of *KRAS* mutations in metastatic lesions from *EGFR* mutant primary tumors (27). Moreover, up to 8–15% NSCLC are diagnosed with multiple lung nodules and can disclose extensive inter-tumor genetic variation in the same patient (28, 29).

In line with these arguments, previous studies investigated and found pathogenic *KRAS* mutations in *EGFR* mutant NSCLC at diagnosis (18–23, 30–32). Percentages of *KRAS* mutation vary widely among studies (range 1.2–10.5%), depending on the broadly different size of the study population (ranging from 58 to 6637 samples), the various analytical sensitivity of the techniques utilized (Sanger, RT-PCR, NGS, ddPCR) and the type of sample analyzed (tissue or cfDNA). Concomitant *KRAS* mutations often involve canonical codon 12 and 13 mutational hotspots and are well known pathogenic mutations which constitutively activate KRAS firing. These mutations could theoretically impact on the response to EGFR inhibitors, due to bypassing the inhibition of EGFR by TKIs. Consequences on clinical responses to *EGFR* TKIs have been investigated in some studies with variable results. In early studies, Takeda et al. and Pao et al. found that *KRAS* mutation is a negative predictor of response to *EGFR*-TKIs in *EGFR* mutation-positive NSCLC patients (33, 34). On the other hand, Benesoma et al. described 3 NSCLC patients with coexistence of *EGFR* and *KRAS* mutations uncoupled from negative response to *EGFR* TKIs (23). More recently, Hong et al. genotyped 58 *EGFR* mutant NSCLC patients before TKI treatment and found that concomitant *KRAS* mutations in cfDNA associated with shorter duration of PFS and OS (18). However, conclusions from these studies were based on small cohorts of patients and other groups reported overlapping clinical outcome in *EGFR* mutant NSCLC patients with or without concomitant *KRAS* mutations (32). These contrasting results could, among other factors, depend on the sub-clonal nature of *KRAS* mutations and their different abundance in the studied patients’ cohorts.

A field relatively less investigated so far involves the prevalence of *KRAS* mutations following treatment and onset of clinical resistance to *EGFR* TKI. Del Re et al. found that 16 out of 33 (48.5%) NSCLC samples studied at progression after *EGFR* TKI had concomitant codon 12 *KRAS* mutations in cfDNA, with percentages of mutated allele ranging from 1–98% (24). However, in this study it was not stated which cut-off has been used for interpretation of ddPCR results. Moreover, accurate assessment of the percentage of *KRAS* mutation in this patient population could be challenging, due to the small number of samples analyzed and the value reported (48.5%) was much greater than previously found by others (18–22, 30–32). In our study by using stringent criteria for interpretation of ddPCR data and analysing a large population of samples (n=104), *KRAS* mutations were rarely found in cfDNA from these patients (2.8%) and had a negative impact on response to TKI and clinical outcome (TTF, PFS, OS) (**Figure 2**). *KRAS* positivity was confirmed in one available matched tumor tissue biopsy at diagnosis. Although this is limited to one patient, results are in-line with a recent study suggesting that *EGFR*-mutated NSCLC patients with *KRAS* mutations detected in tumor before the start of treatment do not benefit from *EGFR* TKIs (22).

It is important to stress that technicalities, such as the cut-off values used to interpret ddPCR results are key to determine the result. In fact, if we lowered the cut-off and considered as *KRAS* mutant even samples with 1–2 positive droplets in cfDNA (n=28), the percentage of *KRAS* mutated samples was much higher (29%). In any case it should be considered that the abundance of *KRAS* mutations was very low, as indicated by the low MAFA values (mean 0.15%, median 0.12%, range 0.016–0.32%), compared with those found in cfDNA from NSCLC patients bearing *KRAS* mutant tumors (mean 8.87%, median 3%, range 0.46–53.7 %) (25). Of regard, we found no prognostic association of borderline *KRAS* mutations in cfDNA with PFS, nor with OS (**Figure 2**).

The main limitation of this study is the relatively limited number of frankly *KRAS* positive patients (n=3), compared with *KRAS* negative patients (n=73). However, our data suggest a potential negative prognostic impact, and confirmed recent reports indicating that *EGFR*-mutated NSCLC patients with additional driver alterations show reduced sensitivity to TKIs. Clearly, our findings should be confirmed in larger series to investigate the impact of *KRAS* mutation detection on clinical decisions, with particular regard to selection of patients for combination treatments, currently under investigation in lung cancer, such as EGFR inhibitor plus chemotherapy or plus antiangiogenic treatment (35).

On the other hand, 36% of plasma samples analyzed at progressive disease were negative for *EGFR* mutation, indicating the possible lack of circulating tumor DNA (ctDNA). This aspect could determine an underestimation of the patients with co-occurring *KRAS* mutations, even though the low presence of ctDNA could be associated with lower tumor burden and better prognosis (36).

Another limitation is represented by the fact that our study did not include systematic analysis of baseline *KRAS* mutation either in plasma or in tissue, which could unravel the multi-clonal character of the tumours. Therefore, we could not draw definitive conclusions on the role of sub-clones in the response to *EGFR* TKI.

We conclude that detection of *KRAS* mutations in cfDNA is rare in *EGFR* mutant patients treated with TKI and these mutations are more likely to be detected in smokers, possibly underlying broader genetic heterogeneity of these tumors compared with those on non-smokers.

## Data Availability Statement

The raw data supporting the conclusions of this article will be made available by the authors, without undue reservation.

## Ethics Statement

The studies involving human participants were reviewed and approved by IOV Institutional Review Board and Ethics Committee (CESC IOV 2020/57). The patients/participants provided their written informed consent to participate in this study.

## Author Contributions

GN contributed to the design of the study, performed the analysis, and wrote sections of the manuscript. JC, LM, and ABB processed the samples and performed the experiments. EZ analyzed the data, and wrote the manuscript. LB, AB, and AM recruited patients, collected, and analyzed clinical data and performed statistical analysis. SI contributed conception and design of the study, wrote the advanced draft of the manuscript revising it critically for intellectual content, and provided approval for publication of the content. All authors contributed to the article and approved the submitted version.

## Funding

This work was funded by IOV intramural research grant 2017 – SINERGIA (to SI and LB). The QX200 ddPCR system (Bio-Rad Laboratories) was purchased through a grant provided by Università degli Studi di Padova, Padova, Italy (2015).

## Conflict of Interest

The authors declare that the research was conducted in the absence of any commercial or financial relationships that could be construed as a potential conflict of interest.
